# Comprehensive study of antimicrobial susceptibility pattern and extended spectrum beta-lactamase (ESBL) prevalence in bacteria isolated from urine samples

**DOI:** 10.1038/s41598-020-79791-0

**Published:** 2021-01-12

**Authors:** Mohammad Javad Gharavi, Javad Zarei, Parisa Roshani-Asl, Zahra Yazdanyar, Masoud Sharif, Niloufar Rashidi

**Affiliations:** 1 Department of Medical Laboratory Sciences, Faculty of Allied Medicine, Iran University of Medical Sciences, Tehran, Iran; 2 Department of Health Information Management, School of Para Medicine, Ahvaz Jundishapur University of Medical Sciences, Ahvaz, Iran; 3 Department of Microbiology, Faculty of Basic Sciences, Shahrekord Branch, Islamic Azad University, Shahrekord, Iran; 4 Department of Medical Laboratory Sciences, Faculty of Allied Medicine, Tehran University of Medical Sciences, Tehran, Iran

**Keywords:** Microbiology, Diseases, Infectious diseases, Bacterial infection

## Abstract

Nowadays, increasing extended-spectrum β-lactamase (ESBL)-producing bacteria have become a global concern because of inducing resistance toward most of the antimicrobial classes and making the treatment difficult. In order to achieve an appropriate treatment option, identification of the prevalent species which generate ESBL as well as their antibiotic susceptibility pattern is essential worldwide. Hence, this study aimed to investigate the prevalence of ESBL-producing bacteria and assess their drug susceptibility in Fardis Town, Iran. A total of 21,604 urine samples collected from patients suspected to have urinary tract infection (UTI) were processed in the current study. The antimicrobial susceptibility of the isolates was tested by the disk diffusion method. The ESBL producing bacteria were determined by Double Disc Synergy Test (DDST) procedure. Bacterial growth was detected in 1408 (6.52%) cases. The most common bacterial strains causing UTI were found *E. coli* (72.16%), followed by *K. pneumoniae* (10.3%) and *S. agalactiae* (5.7%). Overall, 398 (28.26%) were ESBL producer. The highest ESBL production was observed in *E. coli*, followed by *Klebsiella* species. ESBL producers revealed a higher level of antibiotic resistance compared with non-ESBLs. In conclusion, ESBL production in uropathogens was relatively high. Carbapenems and Aminoglycosides were confirmed as the most effective treatment options for these bacteria.

## Introduction

For decades, antibiotics have been used for the treatment of bacterial infections successfully; however, over the past few years, the abuse of antibiotics has led to the emergence of antimicrobial resistance around the world and has become a serious global threat to the public health^1,2^. Recently, it has been reported that approximately 700,000 people worldwide die annually from antimicrobial resistance (AMR) infections and it has been predicted that this number would reach 10 million by 2050^3^.

At present, β-lactam drugs are a key factor in the treatment of bacterial infections worldwide and account for almost 65% of antibiotic usage^4^. They have been classified into six main groups based on the chemical structure of the β-lactam ring which includes Penicillins, Cephalosporins, Cephamycins, Carbapenems, Monobactams, and β-lactamase inhibitors. These drugs block cell wall synthesis by preventing accurate working of the Penicillin-binding protein (PBP), which has a principal role in the synthesis of the bacterial cell wall, and finally leads to cellular death. Nevertheless, it is unfortunate that, in recent years, resistance to this important class of antibiotics is also increasing globally^5^.

Resistance to b-lactams can occur through different mechanisms such as the generation of efflux pumps, changes in the production of outer membrane porins, alterations of PBPs, and the production of β-lactamase for inactivating antibiotics. Of these mechanisms, the production of B-lactamases is the most prevalent source of resistance to β-lactam antibiotics which are produced by both Gram-positive (extracellularly) and Gram-negative (in the Periplasmic space) bacteria. These enzymes are able to make the β-lactam antibiotics inactive by binding covalently to their carbonyl section and hydrolyzing the b-lactam ring thus enabling β-lactam resistance^6,7^.

To date, various β-lactamases have been reported to be generated by diverse microorganisms, including Penicillinases, Extended-spectrum β-lactamases (ESBLs), Cephalosporinases (AmpC), Metallo-β-lactamases (MBLs), and Carbapenemases (KPCs). Among these, ESBL-producing bacteria are very important and have attracted the attention of the scientific community^8^. ESBLs are β-lactamases enzymes with the capability to hydrolyze β-lactam antibiotics containing Penicillins, Aztreonam, as well as the first-, second-, third- and fourth-generation Cephalosporins, while sparing Cephamycins, Moxalactam, and Carbapenems. Further, they are inhibited by β-lactamase inhibitors, such as clavulanic acid, Tazobactam, and Sulbactam^8–10^. ESBLs producing organisms may also induce resistance to some of the none β-lactam antibiotics including Aminoglycosides, Quinolones, and Trimethoprimsulfamath-oxazoles^11^.

Today, the outbreak of infections caused by ESBL producing pathogens is becoming increasingly frequent and has become a world health threat^12^. The plasmid location of ESBL genes contributes to their spread through the horizontal gene transfer among the same and different species of bacteria^13^. The prevalence of ESBL-producing isolates depends on some factors including species, geographic region, hospital/ward, group of patients and type of infection, and extensive overuse of antibiotics^14,15^.

ESBLs are mostly produced by Gram-negative bacilli, especially *Enterobacteriaceae* family^8^. ESBL-producing *Enterobacteriaceae* cause a variety of hospital and community-acquired infections such as bloodstream, wound infections, respiratory tract, and urinary tract infections^16^. Urinary tract infections (UTIs) are very common infectious diseases that occur in a high proportion of the population and are a serious concern in the healthcare system^17^. At present, Carbapenems are selective drugs for the effective treatment of infections caused by ESBL-producing organisms. However, increasing Carbapenem resistance bacteria has also been associated with the use of Carbapenems^18,19^.

Due to expanding antibiotic resistance among bacteria and the high distribution of ESBL producing isolates, the recognition of the prevalent species that produce this enzyme as well as their antibiotic susceptibility pattern is necessary for each community to select the most effective treatment options. Thus, the aim of this study was to determine the prevalence of ESBL-producing bacteria and their antibiotic susceptibility pattern among uropathogens isolated from patients referring to the central laboratory of Fardis Town in Alborz province, Iran.

## Materials and methods

### Study population and samples processing

In the current descriptive cross-sectional study, 21,604 urine samples were aseptically collected from patients suspected to have UTI who were referred to Fardis Town laboratory located in Alborz province, Iran during one year (2018–2019). Positive bacterial growth was detected in urine samples of 1408 (6.52%) patients. The specimens were cultivated on Blood Agar and Eosin Methylene Blue Agar (EMB) medium (Merck, Germany) and incubated at 37 °C for 24 h. Initially, the colonies were counted. In cultures with bacterial counts of > 10^4^ cfu/ml, the specimens were considered as positive, and gram-staining technique was performed. Then, bacterial genus and species were determined by standard biochemical tests.

### Antimicrobial susceptibility testing

Antimicrobial susceptibility of the isolates was performed by the standard Kirby–Bauer disk diffusion method on the Mueller–Hinton agar media (Merck, Germany) using commercially available antibiotic disks (Mast, UK). The diameter of inhibition zone was measured for each antibiotic disk, and the results were defined in accordance with the CLSI guidelines^20^.

### Phenotypic identification of ESBL-producing strains

Detection of ESBL-producing organisms was performed by Double Disc Synergy Test (DDST) method following CLSI recommendations. In this method, first, a suspension was prepared for each pure bacterial isolate according to the 0.5 McFarland turbidity standard and cultured on Mueller–Hinton agar. Fifteen minutes after bacterial cultures, pairs of antibiotic disks containing Ceftazidime (30 μg) with Ceftazidime/Clavulanic acid (30/10 μg), and Cefotaxime (30 μg) with Cefotaxime/Clavulanic acid (30/10 μg) were placed on Mueller–Hinton agar medium center to center, at a distance of 20 mm apart from each other. The plates were incubated for 24 h at 37 °C. Then, the diameter of inhibition zone was measured. According to CLSI guidelines, an increase of ≥ 5 mm in the zone diameter around the clavulanic acid combination disks versus the same disks alone confirmed the presence of ESBL producer strains^20^.

### Ethical considerations

All ethical aspects of this research have been completely observed by the authors. It was approved by Research and Ethics committee of the Iran University of Medical Sciences. All experiments were performed in accordance with relevant guidelines and regulations in Iran. Informed consent was obtained from all participants or their legal guardians before the study. The patient's demographic characteristics were recorded in a questionnaire and their information remained confidential.

### Data analysis

Data were analyzed using descriptive statistics (frequency and frequency percent, average and standard deviation), Chi square statistical test, and Fisher's exact test, hierarchical clustering analysis on SPSS software version 22. The confidence limits for statistical tests were considered as 0.95.

## Results

### Demographic information of the participants

Of a total of 21,604 participants surveyed in the present study, 15,408 (71.3%) were female and 6196 (28.7%) were male. The mean age of subjects was 23.21 ± 33.82 years and their age range was 3 months to 98 years (Table 1).

**Table 1:** Test result age group.

Result		Sex		Age group								Total
		Male	Female	Age ≤ 1	2–5	6–15	16–30	31–45	46–60	61–75	75 < Age	
No bacteria growth		6012	13,837	1659	2047	1834	3257	4923	3228	2299	602	19,849
< 10,000		26	98	58	12	2	19	15	10	3	5	124
*Candida* *albicans* and non-albicans		5	218	1	0	5	73	89	23	18	14	223
**Positive culture**												
Enterobacteriaceae												
*Escherichia coli*		94	922	125	56	83	101	161	196	204	90	1016
*Klebsiella pneumoniae*		15	129	25	3	2	23	32	26	21	12	144
*Citrobacter diversus*		2	21	7	0	0	0	1	3	6	6	23
*Klebsiella oxytoca*		3	10	2	1	0	3	0	1	3	3	13
*Proteus mirabilis*		2	9	6	1	1	1	1	0	1	0	11
*Proteus vulgaris*		1	6	0	4	0	0	2	1	0	0	7
*Citrobacter freundii*		3	2	0	1	0	1	0	0	3	0	5
*Enterobacter aerogenes*		0	4	0	0	0	2	0	1	1	0	4
*Enterobacter agglomerans*		0	3	0	0	0	1	0	0	1	1	3
Streptococcaceae												
*Streptococcus agalactiae*		2	78	0	0	4	19	24	13	14	6	80
*Enterococcus faecalis*		20	31	7	1	4	2	5	12	13	7	51
Non-*Enterococcus bovis*		1	1	1	0	0	0	0	0	0	1	2
Staphylococcaceae												
*Cn Staphylococci*		4	7	1	0	0	2	3	1	3	1	11
*Staphylococcus aureus*		1	8	0	0	1	6	1	0	1	0	9
*Staphylococcus saprophyticus*		1	6	0	0	0	3	3	0	0	1	7
Pseudomonadaceae												
*Pseudomonas aeruginosa*		4	12	2	1	2	0	1	3	2	5	16
Other		0	6	0	0	1	1	2	0	0	2	6
Total		6196	15,408	1894	2127	1939	3514	5263	3518	2593	756	21,604

### The results of urine culture

Positive bacterial growth was detected in urine samples of 1408 (6.52%) patients. Among uropathogens, *E. coli* (1016 cases, 72.16%) was the most commonly isolated species, followed by *K. pneumoniae* (144 cases, 10.3%) and *S. agalactiae* (80 cases, 5.7%). In addition, fungal infection was found in 223 cases. Out of the 1408 positive bacterial cultures, 1255 (89.13%) cases were related to females. As indicated in Table 1, the patients mostly belonged to the age groups of 60–75 years old (283 cases, 20.11%) and 45–60 years old (254 cases, 18%). In both genders, the main infectious strain was *E. coli*, while the proportion of *E. faecalis* infection in males was far higher than in females (13.07–2.47%) (Table 1).

### Minority bacteria

Out of the 1408 culture positive cases, the minimum numbers of isolates were related to*, Proteus mirabilis *(11 cases, 1.6%)*, Proteus vulgaris *(7 cases, 0.5%), *Citrobacter freundii* (5 cases, 0.35%)*, Enterobacter aerogenes *(4 cases, 0.28%), *Enterobacter agglomerans* (3 cases, 0.2%), *Non-Enterococcus bovis* (2 cases, 0.14%)*, Cn Staphylococci *(11 cases, 1.6%)*, Staphylococcus aureus* (9 cases, 0.64%)*, Staphylococcus saprophyticus* strains (7 cases, 0.5%) (Table 1). There isn't any strain producing ESBL among these bacteria.

### Antimicrobial susceptibility profile and ESBL production

#### E. coli

Table 2 indicates the overall antimicrobial susceptibility pattern of *E. coli* for antibiotics tested. It shows that the highest sensitivity was observed with Imipenem (99.2%), Amikacin (97.9%), Meropenem (97.2%), and Nitrofurantoin (92.8%), respectively. Further, the least sensitivity was to Piperacillin (17%) and Ampicillin (19.1%). Out of 1016 *E. coli* isolated, 359 (35.7%) were found to be ESBL producer. With the exception of Amikacin, Imipenem, Meropenem, and Nitrofurantoin, ESBL expression had a significant effect on *E. coli* resistance to other antibiotics tested (Table 2).

**Table 2:** Antibiogram result of *E. coli.*

Antibiotics	Antibiogram result							Statistical significance
	Non-ESBL *E. coli*			ESBL *E. coli*			Total	Pearson chi square/Fisher's exact test
	Sensitive	Intermediate	Resistant	Sensitive	Intermediate	Resistant		
Ciprofloxacin	346	3	99	96	3	165	712	P value = 0.00
Norfloxacin	146	1	41	27	0	72	287	P value = 0.00
Amikacin	635	1	7	352	2	11	1008	P value = 0.046
Ceftazidime	570	2	60	6	0	351	989	P value = 0.00
Cefotaxime	485	5	35	7	1	301	834	P value = 0.00
Ceftriaxone	88	0	22	2	0	53	165	P value = 0.00
Gentamycin	591	5	45	228	3	131	1003	P value = 0.00
Imipenem	582	0	4	332	0	3	921	P value = 0.720
Meropenem	47	0	1	29	0	1	78	P value = 0.734
Nitrofurantoin	610	15	24	330	12	22	1013	P value = 0.138
Cefalexin	480	16	151	5	1	359	1012	P value = 0.00
Ampicillin	191	3	449	2	0	364	1009	P value = 0.00
Nalidixic-acid	333	14	301	56	7	302	1013	P value = 0.00
Co-Trimoxazole	321	10	311	48	2	313	1005	P value = 0.00
Azithromycin	2	0	0	0	0	1	3	*
Colistin	2	0	1	1	0	0	4	
Doxycycline	1	0	1	1	1	0	4	
Chloramphenicol	2	0	0	1	0	2	5	
Piperacillin	1	0	2	0	0	3	6	
Tetracycline	1	0	1	0	0	1	3	
Tobramycin	0	0	0	3	0	2	5	

*No significant statistical test was performed on these antibiotics, due to cases fewer than 6.

#### K. pneumoniae

According to Table 3, *K. pneumoniae* showed the highest rate of sensitivity towards Imipenem (99.3%), followed by Amikacin (95.8%), Meropenem (90%), and Gentamycin (90%), and the least sensitivity to tetracycline (0%), Tobramycin (0%), and Ampicillin (19.1%). From 144 *K. pneumoniae* isolates, 33 (22.9%) were confirmed as ESBL producer. The expression of ESBL resulted in developing resistance to the antibiotics Ceftazidime, Cefotaxime, Ceftriaxone, Gentamycin, Cefalexin, Nalidixic-acid, and Co-trimoxazole.

**Table 3:** Antibiogram result of *K. pneumoniae.*

Antibiotics	Antibiogram result							Statistical significance
	Non-ESBL K. pneumoniae			ESBL K. pneumoniae			Total	Pearson chi square/Fisher's exact test
	Sensitive	Intermediate	Resistant	Sensitive	Intermediate	Resistant		
Ciprofloxacin	73	0	9	15	0	8	105	P value = 0.011
Norfloxacin	24	1	4	5	0	5	39	P value = 0.060
Amikacin	109	0	2	28	0	4	143	P value = 0.023
Ceftazidime	100	0	8	1	0	32	141	P value = 0.00
Cefotaxime	73	0	6	0	0	26	105	P value = 0.00
Ceftriaxone	28	0	3	1	0	5	37	P value = 0.001
Gentamycin	104	0	3	22	1	10	140	P value = 0.00
Imipenem	103	0	0	30	0	1	134	P value = 0.231
Meropenem	8	0	0	1	0	1	10	P value = 0.200
Nitrofurantoin	50	39	22	17	6	10	144	P value = 0.148
Cefalexin	94	1	16	1	0	32	144	P value = 0.00
Ampicillin	5	0	105	0	0	32	142	P value = 0.273
Nalidixic-acid	88	2	21	13	1	19	144	P value = 0.00
Co-Trimoxazole	68	2	41	4	0	29	144	P value = 0.00
Colistin	1	0	0	1	0	1	3	*
Chloramphenicol	0	0	1	1	0	0	2	
Piperacillin	1	0	0	1	0	1	3	
Tetracycline	0	0	1	0	0	1	2	
Tobramycin	0	0	0	0	0	2	2	

*Due to fewer than 5 cases, no statistical test was conducted for these antibiotics.

#### K. oxytoca

The information presented in Table 4 shows that all *K. oxytoca* isolates were reactive towards Imipenem with a sensitivity rate of 100% followed by Amikacin (92.3%). However, none of the isolates was sensitive to Ampicillin (0%). Of the 23 positive urine cultures of *K. oxytoca*, 5 (38.46%) were confirmed as ESBL positive. ESBL-producing isolates were highly resistant to Ceftazidime, Cefotaxime, Norfloxacin, Cefalexin, and Co-trimoxazole.

**Table 4:** Antibiogram result of *K. oxytoca*.

Antibiotics	Antibiogram result							Statistical significance
	Non-ESBL K. oxytoca			ESBL K. oxytoca			Total	Pearson chi square/Fisher's exact test
	Sensitive	Intermediate	Resistant	Sensitive	Intermediate	Resistant		
Norfloxacin	6	1	1	1	0	4	13	P value = 0.050
Amikacin	8	0	0	4	0	1	13	P value = 0.385
Ceftazidime	8	0	0	0	0	5	13	P value = 0.001
Cefotaxime	7	0	1	0	0	5	13	P value = 0.005
Gentamycin	8	0	0	3	0	2	13	P value = 0.128
Imipenem	8	0		5	0	0	13	*
Nitrofurantoin	6	1	1	2	3	0	13	P value = 0.174
Cefalexin	7	0	1	0	0	5	13	P value = 0.005
Ampicillin	0	0	8	0	0	5	13	*
Nalidixic-acid	6	0	2	4	1	0	13	P value = 0.025
Co-Trimoxazole	6	1	1	1	0	4	13	P value = 0.050

*No statistical test was conducted for these antibiotics.

#### S. agalactiae

**Figure 1:**
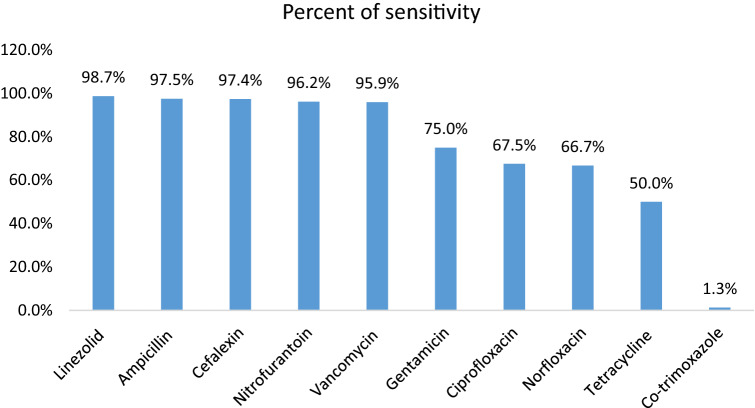
Antibiogram result of *S. agalactiae.*

The antibiotic susceptibility pattern of *S. agalactiae* revealed that the majority of the isolates were sensitive to Linezolid (98.7%), Ampicillin (97.5%), Cefalexin (97.4%), and Nitrofurantoin (96.2%), and the least susceptibility was to Co-trimoxazole (1.3%) (Fig. 1).

#### E. faecalis

**Figure 2:**
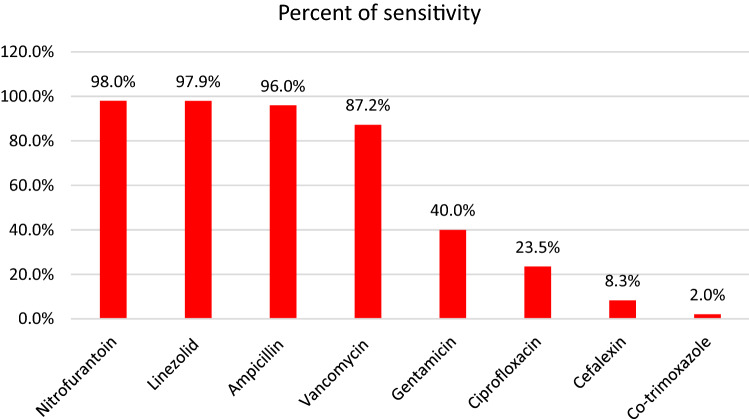
Antibiogram result of *E. faecalis.*

According to the results of antibiogram for *E. faecalis*, Nitrofurantoin (98%), Linezolid (97.9%), and Ampicillin (96%) revealed a superior sensibility for this bacterium. On the other hand, low level of susceptibility was observed in Co-trimoxazole (2%) and Cefalexin (8.3%) (Fig. 2).

#### C. diversus

**Figure 3:**
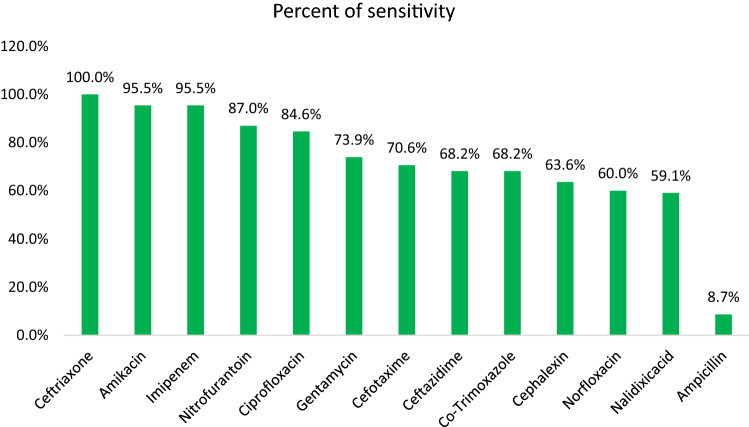
Antibiogram result of *C. diversus.*

In the case of *C. diversus*, Ceftriaxone (100%), Amikacin (95.5%), and Imipenem (95.5%) were found to be most effective antibiotics. However, Ampicillin (8.7%) and Nalidixic-acid (59.1%) showed the least susceptibility rate (Fig. 3). Among 23 *C. diversus* isolates screened for ESBL, one of them was positive for ESBL production which was resistant to antibiotics tested, except of Amikacin and Imipenem.

#### *P. aeruginosa*

**Figure 4:**
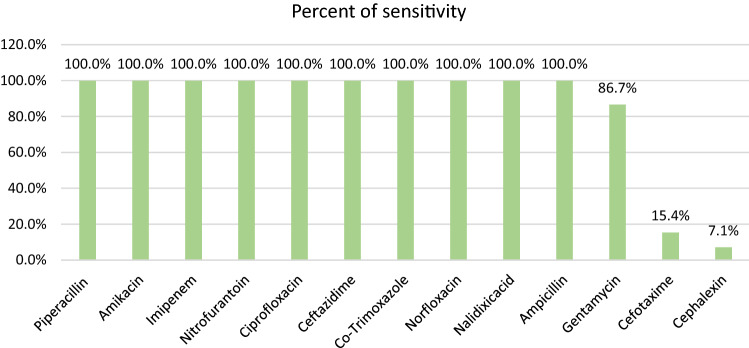
Antibiogram result of *P. aeruginosa.*

As depicted in Fig. 4, the least sensitivity rate for *P. aeruginosa* isolates was against Cefalexin (7.1%) and Cefotaxime (15.4%).

### Hierarchical clustering analysis

**Figure 5:**
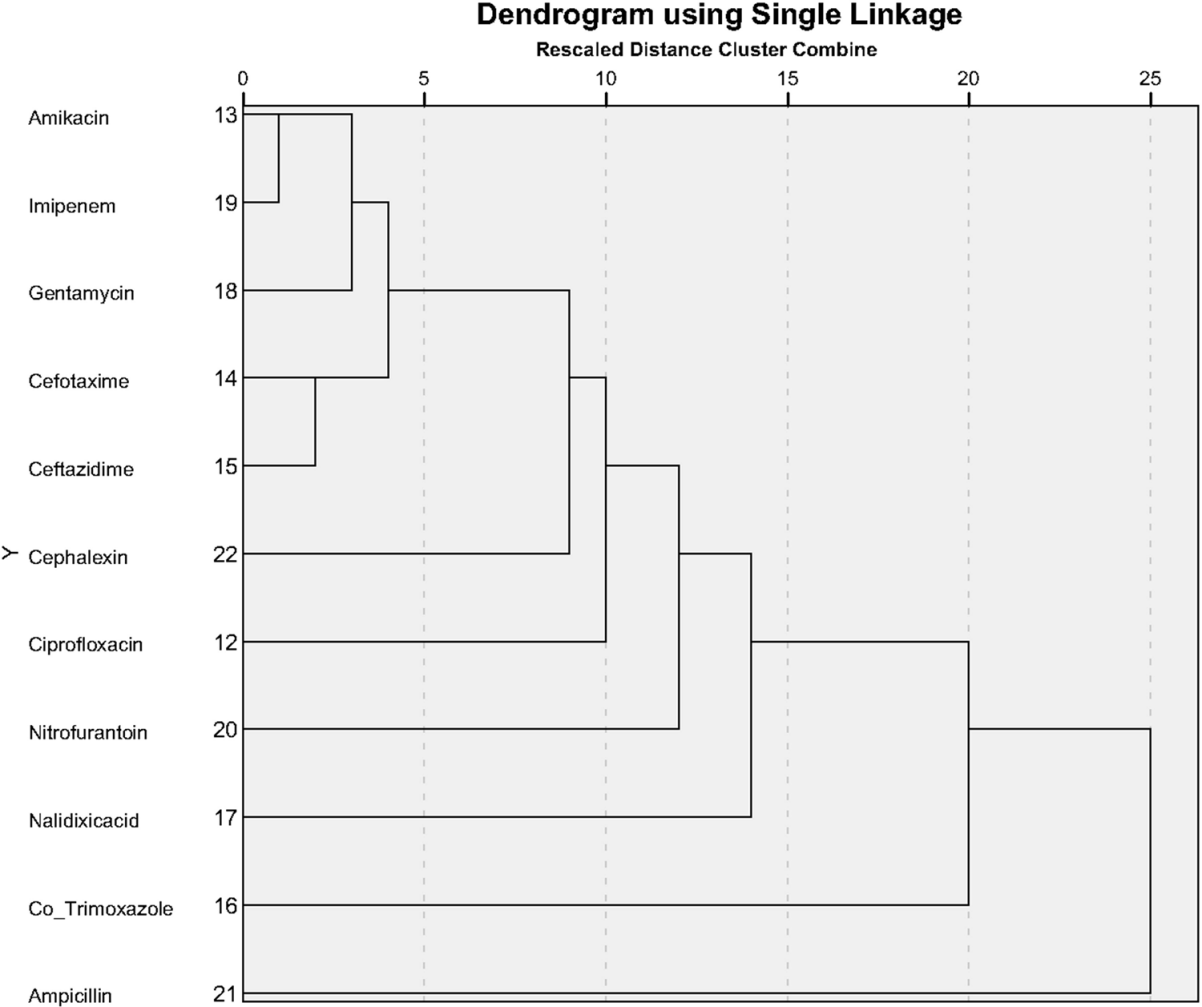
Clustering pattern of antibiotic effect for ESBL-negative bacterial strains.

**Figure 6:**
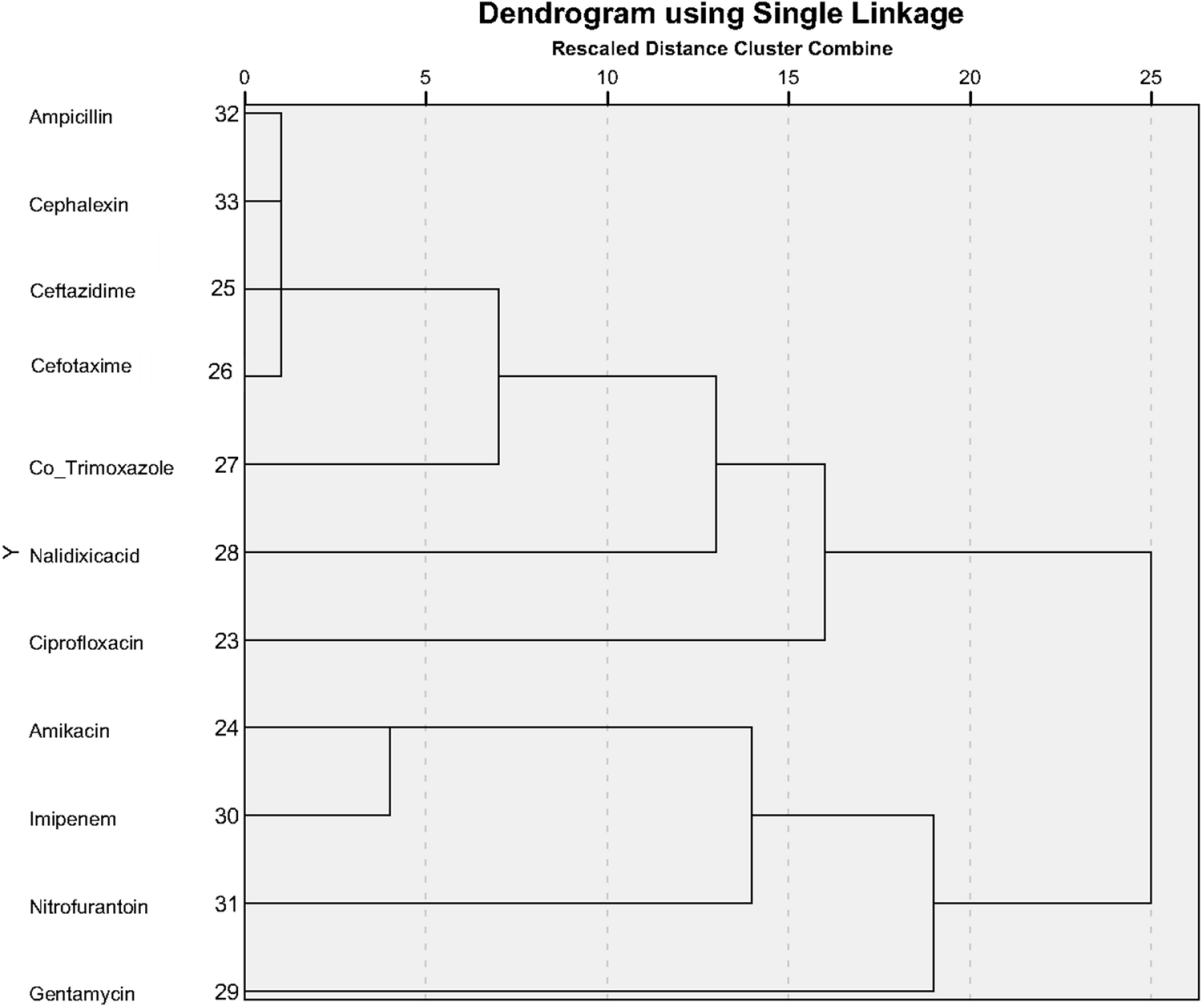
Clustering pattern of antibiotic effect for ESBL-positive bacterial strains.

Using Hierarchical cluster analysis for pattern of antibiotic effect for ESBL-negative/positive bacterial strains, we plotted 11 commonly used antibiotics for three of the bacterial strains producing ESBL (*E. coli*,* K. pneumoniae* and *K. oxytoca*). The goal was to cluster antibiotics with similar efficacy. Clustering was done by the nearest neighbor method and Minkowski measure. Figures 5 and 6 (Dendrogram plots) show the clustering of antibiotics with similar effects for ESBL-negative and ESBL-positive bacterial strains.

As depicted in Fig. 5, the two antibiotics Amikacin and Imipenem were most similar in their effect on ESBL-negative bacterial strains.

As depicted in Fig. 6, the antibiotics Ampicillin, Cephalexin, Ceftazidime, and Cefotaxime had the most similarity in their effect on ESBL-positive bacterial strains.

## Discussion

Urinary tract infection (UTI) is the second common infectious disease throughout the world caused by a wide range of microbial pathogens^21^. In the present work, from 21,604 suspected UTI patients, 1408 (6.52%) uropathogenic bacteria containing different species of Gram-negative and Gram-positive bacteria were isolated. The majority of the isolates were obtained from females 1255 (89.13%). This finding is supported by other studies reporting a higher rate of UTI prevalence in female patients compared to males^14,22^. These studies suggest that females are more at risk of developing infection by uropathogens which is due to their anatomical structure^23^. In terms of age, it was found that the most frequently uropathogens were related to the age groups of 60–75 (20.11%) and 45–60 (18%) years old. These outcomes agree with previous studies in which the incidence of UTIs was higher among elderly patients^24,25^.

The members of *Enterobacteriaceae* family especially *E. coli* and *Klebsiella* spp. are identified as vital causative agents of UTIs as they possess a number of factors including adhesion, pilli, fimbrae, and P1 blood group genotype receptor, which contribute to the attachment of bacteria to the urothelium^26^. In this regard, here the predominant urinary isolates were *E. coli* 1016 (72.16%) followed by *K. pneumoniae* 144 (10.3%). These results are in line with the earlier studies conducted by other researchers^27–29^. In addition, we found *S. agalactiae* 80 (5.7%) as the most frequently isolated Gram-positive bacterium involved in UTI.

At present, increasing *Enterobacteriaceae* producing ESBLs is a global healthcare concern because of high antibiotic resistance and the restricted treatment options^30^. In this survey, ESBL production was found in 28.26% (398/1408). In line with this result, some researchers reported the rate of ESBL production as 29% and 30.23%^31,32^. In contrast, an earlier study revealed that 11.75% of uropathogens were ESBL positive^33^ which is lower than our result. Elsewhere, the researcher reported 55.4% for ESBL production rate which is larger than ours^34^. These differences may be due to geographical area, time, and the diagnostic technique used^32^.

Among the ESBL producers, the highest rate was observed in *E. coli* (35.7%), followed by *Klebsiella* spp. (22.7%) and *C. diversus* (4.34%). This is in accordance with other studies in which among various ESBL isolates, *E. coli* species was the most prominent isolates followed by *Klebsiella* spp. On the other hand, the minimum ESBL isolates were related to *Citrobacter* spp.^35–37^. In contrast, several studies reported the highest ESBL production among *K. pneumoniae* followed by *E. coli* which do not match our results^38–40^, since we found *E. coli* as the predominant ESBL producer.

The antibiotic susceptibility testing by commonly prescribed antibiotics was accomplished for the most frequent pathogens found in our study. In determining the antibiotic susceptibility patterns of *E. coli*, a high level of sensitivity to Imipenem (99.2%), Amikacin (97.9%), Meropenem (97.2%), and Nitrofurantoin (92.8%) was observed, while the least sensitivity was related to Piperacillin (17%) and Ampicillin (19.1%). Most studies on the antibiotic susceptibility of urinary pathogens around the world have found similar results. For example, in Ahmed et al.’s study, *E. coli* showed high resistance to Ampicillin and Piperacillin, and low resistance rates against Meropenem, Amikacin, and Nitrofurantoin^41^. In another study by Mohammed et al., isolated *E. coli* was highly sensitive to Amikacin, Imipenem, and Meropenem, and extremely resistant to Ampicillin^42^. Shakibaie et al. exhibited that uropathogenic *E. coli* were 100% sensitive to Imipenem and Meropenem, and 94.4% to Amikacin, while all of them were resistant to Ampicillin^43^. Another study conducted by Koshesh et al. revealed a low rate of resistance to Imipenem for *E. coli* uropathogens^44^. In Khan et al.'s study, *E. coli* showed 96.2% susceptibility to Imipenem, 85.1% to Amikacin, and 72.6% to Nitrofurantoin^45^.

In the case of *P. aeruginosa*, we found it 100% sensitive to 10 antibiotics from 13 antibiotics tested. Its minimum sensitivity was observed to Cefalexin (7.1%) and Cefotaxime (15.4%). A similar study by Shah et al. reported that Imipenem, Piperacillin/Tazobactam, and Amikacin with a minimum resistance rate were the most effective antibiotics against *P. aeruginosa* in UTI patients^46^. In contrast, a study by Abdollahi et al. showed that *P. aeruginosa* isolated from UTI patients were highly resistant to Ampicillin (100%), Gentamicin (66.7%), and Nalidixic-acid (66.7%)^47^. On the other hand, we found no resistance to Ampicillin and Nalidixic-acid, while resistance to Gentamicin was very low (13.3%).

*S. agalactiae* isolates in our study were highly sensitive to Linezolid, Ampicillin, Cefalexin, and Nitrofurantoin. In agreement with our findings, Shayanfar et al. found high susceptibility to Ampicillin (96%), Nitrofurantoin (95.5%), Vancomycin (95%), and Norfloxacin (96.5%). They also found the most resistance to Tetracycline (81.6%) and Co-trimoxazole (68.9%)^48^, while in our study the most resistance was seen to Co-trimoxazole (98.7%) followed by Tetracycline (50%). Another study conducted by Tayebi et al. showed that all *S. agalactiae* urinary isolates were sensitive to Linezolid and Vancomycin, 99.6% to Ampicillin and nitrofurantoin, which is in line with our results. Nevertheless, they showed 12% sensitivity to Tetracycline which was lower than ours (50%)^49^.

Regarding *E. faecalis* isolates, we found Nitrofurantoin (98%), Linezolid (97.9%), Ampicillin (96%), and Vancomycin (87.2%) as the most effective antibiotics. Similar to our results, Goel et al. reported that *E. faecalis* isolated from UTI patients was highly sensitive to Linezolid (100%), Nitrofurantoin (86%), Vancomycin (77.1%), Gentamicin (67.3%), Ampicillin (63.9%), and Ciprofloxacin (31.2%)^50^. Their Gentamicin susceptibility was more than ours. Further, Akhter et al. demonstrated considerable sensitivity to Vancomycin (100%), Nitrofurantoin (85.72%), and Ciprofloxacin (23.81%), which is similar to this study's findings. They also showed 28.58 sensitivity rate for Gentamicin and Co-trimoxazole, which is different with our results^51^.

Concerning *C. diversus* isolates, they were highly sensitive to Ceftriaxone (100%), Amikacin (95.5%), and Imipenem (95.5%). Sami et al. found that *Citrobacter* species isolated from UTI patients were sensitive to Imipenem (100), Amikacin (85.2%), and Gentamicin (77.4%), which is consistent with our findings. In addition, they found sensitivity to Nitrofurantoin (66.1%), Ciprofloxacin (56.2%), Ceftriaxone (50.9%), and cefotaxime (43.3%) which are lower than ours^52^.

Considering the results of antibiogram test for *K. oxytoca* isolates, the greatest sensitivity was related to Imipenem (100%) and Amikacin (92.3%). This agrees with Razzaque et al.'s study which found Imipenem (94.7%) and Amikacin (92.3%) as the most effective antibiotics for *K. oxytoca* urinary isolates. They also found sensitivity to Nitrofurantoin (84.6%) and Gentamicin (65.5%), which are different to our results^53^.

Depending on the ESBL-positive or negative bacteria, this study suggested that ESBL-producing isolates had higher resistance to some antibiotics tested compared to non-ESBL producers. For example, ESBL-positive isolates of *E. coli* were more resistant to antibiotics tested except Amikacin, Imipenem, Meropenem, and Nitrofurantoin. ESBL-producing *K. pneumoniae* presented a higher resistance rate to Ceftazidime, Cefotaxime, Ceftriaxone, Gentamycin, Cefalexin, Nalidixic-acid, and Co-trimoxazole. Also, in *C. diversus* isolates, ESBL production caused resistance to antimicrobial agents except for Amikacin and Imipenem. Regarding *K. oxytoca*, it led to resistance to Ceftazidime, Cefotaxime, Norfloxacin, Cefalexin, and Co-trimoxazole.

In line with these outcomes, a study carried out by Poovendran et al. indicated that resistance to Tetracycline, Amikacin, Ampicillin, Tobramycin, and Norfloxacin was comparatively higher among uropathogenic *E. coli* ESBL-producer than non-ESBL producer. Nevertheless, both groups of isolates were 100% sensitive to Imipenem^54^. Furthermore, Albu et al. found that ESBL-producing *E. coli* and *K. pneumoniae* isolates were more resistant to most of the antibiotics tested compared to non-ESBLs, except for Amikacin for E. coli and Imipenem for *K. pneumoniae*, which had lower resistance rates than non-ESBLs^55^. Further, another study by Abayneh et al. indicated that ESBL-positive bacteria had higher resistance to most of the antimicrobial agents tested, while in both ESBL-producing and non-ESBL-producing isolates, no resistance was observed toward Imipenem and resistance to Amikacin was low^56^. Additionally, we used Hierarchical clustering analysis based on the antibiogram pattern of ESBL-negative/positive *E. coli*, *K. pneumoniae* and *K. oxytoca* strains for clustering antibiotics with similar effects. In the case of ESBL positive strains, the results showed that the effectiveness of Cephalexin, Cefotaxime, and Ceftazidime was similar to that of Ampicillin, an antibiotic which had poor effect, and about the ESBL-negative strains, Amikacin and Imipenem demonstrated the most similarity in their efficacy.

In conclusion, the current study indicated a significant rate of infection with ESBL-producing Gram-negative bacilli among UTI patients. The ESBL production was found predominantly among *E. coli* followed by *Klebsiella* spp. An intensifying level of resistance to various classes of antimicrobial agents was observed among ESBL producers compared with non-ESBLs.

Hierarchical cluster analysis on the antibiogram pattern showed that in ESBL-positive bacterial strains, the efficacy of Cephalosporins; Cephalexin, Cefotaxime, and Ceftazidime was similar to that of Ampicillin, an antibiotic that had very little effect. With respect to the results of this research and similar investigations worldwide, Carbapenems and aminoglycosides were confirmed as the best options for antibiotic therapy against the ESBL-producing isolates. On the other hand, Penicillins and Co-trimoxazole are not recommended in the treatment of these bacteria; also, the administration of Cephalosporins should be limited. In conclusion, given the rise of antibiotic resistance and the high prevalence of ESBL production in Gram-negative bacteria, plus the importance of this issue in the field of treatment and public health and the costs associated with it, precise infection control and careful monitoring of antibiotic administration is crucial. Thus, routine screening of ESBL-producing isolates before prescribing antibiotics is recommended to prevent prolonged and inappropriate use of antibiotics and therapeutic failures.
